# Associations between Physical Function, Bone Density, Muscle Mass and Muscle Morphology in Older Men with Sarcopenia: A Pilot Study

**DOI:** 10.3390/medicina57020156

**Published:** 2021-02-09

**Authors:** Asta Mastavičiūtė, Justina Kilaitė, Donatas Petroška, Arvydas Laurinavičius, Marija Tamulaitienė, Vidmantas Alekna

**Affiliations:** Faculty of Medicine, Vilnius University, 03101 Vilnius, Lithuania; justina.kilaite@mf.vu.lt (J.K.); donatas.petroska@mf.vu.lt (D.P.); arvydas.laurinavicius@mf.vu.lt (A.L.); marija.tamulaitiene@mf.vu.lt (M.T.); vidmantas.alekna@mf.vu.lt (V.A.)

**Keywords:** sarcopenia, muscle mass, bone mineral density, physical performance, muscle morphology

## Abstract

*Background and Objectives:* It is thought that muscle and bone interact only on a biomechanical level, however, some research is now emerging that links bone and muscle on a cellular level. The aim of this study was to explore associations between physical function, muscle mass and bone density in community-dwelling elderly men with sarcopenia. A secondary goal was to analyze if muscle morphology was associated with bone density and physical functioning. *Materials and Methods:* Body composition was measured by dual-energy X-ray absorptiometry (DXA). Bone density was evaluated according to WHO criteria. Sarcopenia was diagnosed according to European Working Group on Sarcopenia in Older People (EWGSOP) criteria: low muscle mass and low muscle strength or low physical performance. Microbiopsy of *musculus vastus lateralis* was performed with a disposable muscle microbiopsy system. The perimeter and cross-sectional area of muscle fibers were calculated using image analysis software in whole slide images; type of fibers and their distribution were evaluated as well. *Results:* A total of 151 men, 60 years or older were included in this study. Mean age of the subjects was 72.9 ± 8.02 years. Sarcopenia was diagnosed in 45 (29.8%) men. Multiple significant correlations were found between bone mineral density, lean mass, appendicular lean mass, arm and leg lean mass, gait speed, balance test and handgrip strength in sarcopenic men. Lean mass was associated with femoral neck BMD (bone mineral density; r = 0.418, *p* = 0.006) and handgrip strength (r = 0.553, *p* < 0.001). In the sarcopenia group, 25 muscle biopsies were examined. In 9 sarcopenic men with T-scores equal or below −2.5, the muscle fiber area had a significant correlation with the balance test (r = 0.73, *p* = 0.025). *Conclusions:* In men with sarcopenia, low lean muscle mass was associated with low femoral neck BMD and low muscle strength. In sarcopenic men with osteoporosis, low muscle fiber area was associated with low scores in a balance test.

## 1. Introduction

In 1989, Rosenberg described age-related loss of lean body mass as sarcopenia [1]. Although there is no one unifying definition of sarcopenia, all current definitions include low muscle mass and function, be it either muscle strength or physical performance, or both [2]. Depending on the definition used, the prevalence of sarcopenia varies from 9.9 to 40.4% in community-dwelling older adults [3]. Sarcopenia is associated with such negative outcomes as falls, increased hospitalization, functional decline and death [4,5].

One of the hallmark components of sarcopenia is low muscle mass. Muscle consists of different types of fibers (type I and type II). With aging, the number and size of fibers, especially type II, decreases [6,7]. Furthermore, age-related changes lead to fiber atrophy [8]. This in turn may lead to muscle atrophy and reduced muscle mass. 

Bone mineral density (BMD) is used to assess bone density. Furthermore, BMD is a component of body composition measurements [9]. Low BMD is a risk factor of low-energy fractures which are very characteristic for osteopenia or osteoporosis [10]. Positive associations between BMD, lean muscle mass, and fat mass have been found [11,12,13]. It is believed that bone and muscle interact only on biomechanical level, meaning that the force produced by muscles affects bone strength and density [14]. Furthermore, a relationship exists between bone density, muscle strength and physical performance as physical activity affects bone and muscle metabolism [12]. However, a new theory is now emerging that links bone and muscle transformations at the biochemical level via endocrine and paracrine systems [15]. Therefore, muscle and bone could be more related than previously thought and structural changes in one tissue could affect the other tissue as well. 

The link between sarcopenia and physical performance is known as some operational definitions of sarcopenia use it to assess muscle performance [16]. In addition, the impact of osteoporosis on physical functioning has also been confirmed [17]. The term osteosarcopenia is being used to describe the condition when sarcopenia and osteoporosis are seen together in one person [18]. However, there are a limited number of studies looking at the relationship between sarcopenia and osteoporosis on a structural level, and their association with physical function. 

Therefore, the aim of this study was to explore associations between muscle mass, bone density and physical function in community-dwelling elderly men with sarcopenia. After that, we took muscle biopsies and analyzed if muscle morphology was associated with bone density and physical functioning. 

## 2. Materials and Methods

### 2.1. Study Population

Ambulatory, community-dwelling men, aged 60 years and older, who attended the National Osteoporosis Center in Vilnius, Lithuania, were invited to participate in this study. Exclusion criteria were: an objection to any procedure, large dose of radiation received over the past 12 months, malignant tumors of various localizations, mental disorders, muscle diseases (hereditary and inflammatory), and current/past use of any medications likely to affect muscle, bone and fat metabolism. This study protocol was approved by the Lithuanian regional biomedical research ethics committee (No. 158200-03-208-75; date: 8 March 2011). All subjects gave their written informed consent prior to the enrolment to the study. Height and weight were measured and body mass index (BMI) was calculated by dividing weight by height squared. The number of concomitant illnesses and medications were collected from medical records. 

### 2.2. Assessment of Body Composition

Body composition was assessed by dual-energy X-ray absorptiometry (iDXA, GE Lunar, Madison, WI, USA). Total body fat mass, total lean mass, appendicular lean mass, and arm and leg lean mass were measured. Fat distribution was assessed using the android and the gynoid region of interest defined by the manufacturer, located respectively in the abdominal and in the hip area. Appendicular skeletal muscle mass calculation was based on the sum of muscle mass in the limbs. Bone mineral density (BMD) and bone mineral content (BMC) were evaluated from total body measurements. Lumbar spine (L1–L4, in the anterior-posterior direction) and left femoral neck BMD were also measured. Bone mineral density was evaluated according to World Health Organization criteria: a T-score between −1.0 and −2.5 was categorized as osteopenia and a T-score equal or below −2.5 as osteoporosis [19]. 

### 2.3. Definition of Sarcopenia

In this research, a diagnosis of sarcopenia was confirmed according to the European Working Group on Sarcopenia in Older People (EWGSOP), with its criteria proposed in 2010: low muscle mass and low muscle strength or low physical performance [16]. A skeletal muscle mass index was calculated by dividing appendicular skeletal muscle mass by the subjects’ height squared. The cut-off of 7.26 kg/m^2^ for skeletal muscle mass index in men was used to determine low muscle mass [20]. Muscle strength was assessed by handgrip strength which was measured using a mechanical handheld dynamometer (Presision, Druck, Germany). Participants were asked to sit upright and hold the dynamometer in their non-dominant hand. Men were asked to squeeze the dynamometer three times as tightly as possible, and the highest result was recorded. The cut-off of 30 kg was used as the diagnostic criterion for low muscle strength [21]. Both devices, the DXA machine and the dynamometer, were calibrated following the manufacturer’s instructions. Physical performance was evaluated by short physical performance battery (SPPB) composed of three tests: balance, 4 m gait speed and chair stand [22,23]. The balance test included tandem, semi-tandem and side-by-side standing. Participants were timed until they were no longer able to continue standing or 10 s had passed. To assess speed, participants were asked to walk 4 m at their usual pace. For the chair stand test, participants were asked to fold their arms across their chest and stand up from the chair five times as quickly as possible. Gait speed equal or lower to 0.8 m/s was used as diagnostic criterion for sarcopenia [21]. 

### 2.4. Muscle Biopsy

Microbiopsies were performed in men with sarcopenia who agreed to this procedure. For microbiopsy, each subject was asked to lie down on the medical functional bed with the thigh area uncovered. The skin of the microbiopsy area (*musculus vastus lateralis*, about 15 cm above the patella) was disinfected and local anesthesia with lidocaine solution was applied. A piece of muscle was taken using a disposable muscle microbiopsy system with 14–16 G and 10 cm long needle for muscle microbiopsy (Bard Monopty Disposable core biopsy instrument, Franklin Lakes, NJ, USA). A sterile dressing was applied on the puncture site. After the muscle biopsy sample was obtained, it was kept surrounded by melting ice and transported to the laboratory immediately. In the laboratory, unfixed muscle was frozen for 30 sec and kept on cork in liquid nitrogen. Thereafter, all collected samples were cryosectioned at 10 μm of thickness for hematoxylin/eosin (H/E), adenosine triphosphatase (ATP) histochemical and 3 μm of thickness for immunohistochemical merosin (Monoclonal Mouse Anti-Human Merosin Laminin Alpha 2 Chain) staining. Immunostaining procedure was done using this protocol: before staining, procedure tissue was fixed in cold acetone for 10 min then the slide was washed with TBST buffer; incubation for 30 min at room temperature with primary antibody (Merosin, Leica Systems, clone: Mer3/22B2, dilution: 1:15) and incubation for 30 min at room temperature in EnVisionFLEX/HRP was performed; after that, counterstaining of sections in hematoxylin and mount coverslipping was done. 

In H/E stained slides, cases were selected as having myofibers by experienced pathologists using a light microscope. Further selected cases were stained for ATP and merosin and these slides scanned for digital pictures. The perimeter of myocytes marked by merosin additionally were annotated manually in digital slides at ×200 magnification in order to escape the evaluation of artefactually damaged or not perpendicularly orientated (e.g., tangentially, longitudinally) myofibers. There were annotated and analyzed at 50–455 myofibers per case. Manual annotation in digital slides was selected in order to mark undamaged myofibers or the only few myofibers with minor microvacuolization and myofibers with only perpendicular orientation. Annotated cross-sectional areas of muscle fibers were once analyzed and calculated using the Aperio software (Image Scope, Buffalo Grove, IL, USA) Membrane algorithm. The samples of skeletal muscles were also stained with adenosine triphosphatase and type I and type II fibers, and their distributions were evaluated by Aperio software as well.

### 2.5. Statistical Analysis

Statistical analysis was performed using IBM SPSS Statistics Windows software version 18 (IBM, New York, NY, USA). All data were expressed as mean, standard deviation (SD) or frequencies (number, percentage), as appropriate. Where the data were skewed or not normally distributed, median and interquartile ranges were used. Distribution of continuous variables was assessed by the Shapiro–Wilk test. Normally distributed dichotomous categorical and continuous variables were compared using independent sample T-test. Mann–Whitney U test was used to compare independent groups for non-parametric dichotomous variables. Chi-square test was used to compare categorical variables. Relationships between continuous variables were examined using Spearman’s correlation. A correlation above 0.81 was considered as excellent, between 0.61 and 0.80 very good, between 0.41 and 0.6 as good, between 0.21 and 0.4 acceptable, and less than 0.20 was considered insufficient [24]. The level of significance (*p*-value) of < 0.05 was considered to be statistically significant.

## 3. Results

### 3.1. Participant Characteristics

A total of 151 men participated in this study. According to EWGSOP criteria, sarcopenia was defined in 45 (29.8%) men. Basic descriptive characteristics of the study population are shown in Table 1.

Sarcopenic men were older than non-sarcopenic men, with lower weight and BMI as well. Moreover, the mean BMI score in all groups belongs to pre-obesity status. In addition to this, fat mass, lean mass, appendicular lean mass, arm lean mass, leg lean mass, skeletal muscle mass index, handgrip strength and gait speed were lower in sarcopenic men. Whole body, femoral neck BMD and whole body, femoral neck T-score and BMC were higher in non-sarcopenic men; however, no difference was found between lumbar spine BMD and T-score in non-sarcopenic and sarcopenic men. The largest proportion of men with T-score equal to or below −1.0 was found in the sarcopenic group, where almost 87% of men could be classified as having low BMD. 

### 3.2. Relationship between Bone Density and Body Composition

When analyzing bone density and body composition in older men with sarcopenia, it was found that multiple associations exist between bone density and components of body composition (Table 2).

As shown in Table 2, acceptable and good positive correlations were found between bone density, lean mass parameters and some physical performance measures. Furthermore, positive correlations were also found between fat mass and whole body BMD (r = 0.372, *p* = 0.014). In addition, gynoid fat mass was positively associated with whole body T-score (r = 0.317, *p* = 0.038). An acceptable correlation was found between lumbar spine T-score and SMMI (r = 0.323, *p* = 0.035).

### 3.3. Association between Physical Performance, Bone Density and Lean Mass

There were no correlations between physical performance measured with 5 chair stands test and bone density. Gait speed had positive correlation with appendicular lean mass (r = 0.323, *p* = 0.037) and arm lean mass (r = 0.327, *p* = 0.035). Moreover, the balance test was associated with appendicular lean mass and arm lean mass as well (r = 0.347, *p* = 0.024; r = 0.384, *p* = 0.012, respectively), as with SMMI (r = 0.334, *p* = 0.031). No associations were found between leg lean mass and gait speed or balance, and between 5 chair stands test and body composition parameters.

### 3.4. Relationship between Strength, Bone Density and Lean Mass

Handgrip strength had positive associations with femoral neck BMD, whole body T-score and BMC (r = 0.386, *p* = 0.011; r = 0.419, *p* = 0.005; r = 0.387, *p* = 0.011, respectively). Furthermore, handgrip strength had positive correlations with femoral neck T-score (r = 0.443, *p* = 0.003). Skeletal muscle mass index also had an acceptable positive correlation with handgrip strength (r = 0.342, *p* = 0.027). The study results showed that in sarcopenic men, handgrip strength was positively associated with lean mass (r = 0.553, *p* < 0.001), appendicular lean mass (r = 0.612, *p* < 0.001), arm lean mass (r = 0.6, *p* < 0.001) and leg lean mass (r = 0.572, *p* < 0.001).

### 3.5. Association between Muscle Morphology, Bone Density, Body Composition and Physical Function

**Figure 1:**
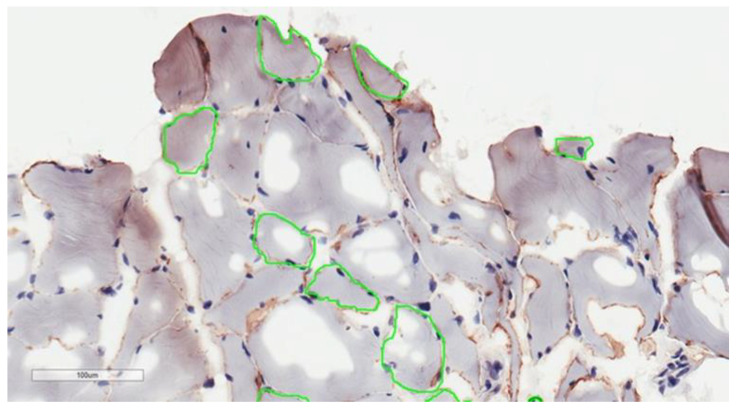
Microscopical view of the skeletal muscle biopsy stained with merosin and with manual annotations (green line) of sarcolemma at visually the most perpendicularly orientated cross sectioned and at least artefactually damaged myocytes.

Out of 45 men with sarcopenia, 38 agreed to undergo a procedure of muscle biopsy. A total of 8 out of those men had contraindications for biopsy: 2 were taking anticoagulants at the time and 6 had a confirmed case of arrhythmias. Out of the 30 muscle microbiopsies performed, 25 samples were informative, because 2 microbiopsies had insufficient samples and 3 had higher than the accepted count of altered fibers. A picture of a muscle morphology sample can be seen in Figure 1.

Using calculations from Aperio software Membrane type algorithm, annotated muscle fiber length, on average, was 217.47 ± 25.22 µm, the smallest length was 183.47 µm and the longest—255.83 µm. Fiber cross-sectional area varied from 139 to 6116 µm^2^. Average fiber cross-sectional area was 2446 ± 608.87 µm^2^. The number of type I fibers counted in ATP staining sections by software was 168.75 ± 89.35 and of type II fibers, it was 126.25 ± 74.87. Type I fibers made up 57.28% of all fibers.

There were no significant correlations between muscle fiber length and whole body, lumbar spine or femoral neck BMD or bone mineral content. Muscle fiber area did not correlate with bone density either. No correlations between muscle fiber length or area and T-score in whole body, lumbar spine or femoral neck were found. Body composition parameters, such as fat mass, android and gynoid fat mass, lean mass, appendicular lean mass, arm and leg lean mass were also not associated with muscle fiber length or muscle fiber area. Moreover, no correlations were found between muscle morphology and physical performance or muscle strength.

In nine sarcopenic men with T-score <−2.5, muscle fiber area had a good positive correlation with results of the balance test (r = 0.73, *p* = 0.025). No other associations were found between muscle fiber length or area and bone density, body composition, physical performance or muscle strength. In the other 16 men whose T-score was >−2.5, no correlations were found between muscle morphology, BMD, body composition, physical performance and muscle strength.

## 4. Discussion

The results of our study showed that associations between body composition, bone density, physical performance and muscle strength are intertwined within the groups. Multiple significant correlations were found between bone mineral density, lean mass, appendicular lean mass, physical performance and handgrip strength. Furthermore, an association was found between muscle morphology and physical performance.

In this study, we found that lean mass, appendicular lean mass, arm and leg lean mass and skeletal muscle mass index, which is calculated by dividing appendicular lean mass from subject’s height squared, had positive associations with femoral neck and whole body BMD and T-score. SMMI was also associated with lumbar spine T-score as well. Some of these results are similar to those found by Verschueren et al. [25]. They found that higher appendicular lean mass and skeletal muscle mass index were associated with higher BMD scores in whole body, femoral neck, total hip and lumbar spine. It should be noted that their study included not only older, but middle-aged men as well. Moreover, in the Hertfordshire Sarcopenia Study, it was found that appendicular lean mass was associated with whole body BMD, lumbar spine BMD and femoral neck BMD [26]. Turning to body composition association with T-score, in a Swiss study, men with sarcopenia, which was characterized as skeletal muscle mass index lower or equal to 7.26 kg/m^2^, had significantly lower femoral neck T-scores compared to men who had higher skeletal muscle mass index [27]. Furthermore, Intriago and colleagues found that in older adults with osteoporosis lean mass, appendicular lean mass and SMMI were lower than in older adults without sarcopenia [28]. However, the majority of participants in that study were women. Conversely to the previously mentioned findings, one cross-sectional study in China found no differences between skeletal muscle mass index in older adults and T-score [29]. It should be noted that in this study, BMD was not measured by the DXA, but by the quantitative ultrasound at calcaneus. Furthermore, this study included both older men and women. We not only found associations between bone density and lean mass, but with fat mass as well. Whole body BMD was correlated with fat mass and whole body T-score was associated with gynoid fat mass. A cross-sectional study in non-sarcopenic older women found that fat mass was associated with femoral neck, hip and lumbar spine BMD [30]. Contrary to this, a cross-sectional study in older Korean women found no relationship between fat mass and femoral neck or hip BMD [31].

In our study, gait speed and balance test were associated with femoral neck T-score, and the balance test alone had correlations with BMC and whole body T-score in older men with sarcopenia. Furthermore, gait speed and balance test were related to appendicular lean mass and arm lean mass. Balance test and SMMI were also correlated. Surprisingly, leg lean mass did not correlate with gait speed or balance test, which is odd knowing that the legs play a major role in gait speed and balance test assessment.

Handgrip strength, which acts as a proxy for muscle strength assessment, in our study was associated with bone density and body composition parameters. Femoral neck BMD and T-score as well as whole body T-score and BMC were correlated with handgrip strength. In older Chinese adults, higher handgrip strength was associated with lower risk of osteoporosis [29]. Although in older Chinese women with osteoporosis, handgrip strength was correlated with femoral neck, hip and lumbar spine BMD, in the same study such correlations were not found in the male group [32]. As mentioned earlier, handgrip strength is also associated with lean mass, appendicular lean mass, arm and leg lean mass and SMMI. In Sweden, in community-dwelling older men with moderate and severe sarcopenia, a positive correlation was found between SMMI and handgrip strength [33]. In older adults without sarcopenia, higher fat free mass is associated with higher handgrip strength [34]. No correlations were found between muscle morphology (muscle fiber length and muscle fiber area) and bone density. However, in the Hertfordshire Sarcopenia Study, unadjusted analysis revealed associations between type I fiber area and femoral neck BMC, whereas after the adjustment for age and height, these associations were not significant [26]. Furthermore, they found that type II fiber area was associated with femoral neck BMD and BMC before and after the adjustment. In addition, they did not observe any significant correlations between type I or II fiber areas and whole body or lumbar spine BMD or BMC. Other studies conducted in osteoporotic women also found that total hip and femoral neck BMD values correlated with the percentage of type II fiber atrophy [35]. In this study, we found that men with sarcopenia and T-scores below −2.5 muscle fiber area had a positive correlation with the balance test performance. In our opinion, we are the first to report on this association. Nonetheless, the sample in which this connection was found was very small and more in depth studies are needed to confirm this association.

We also found that in this study, a larger proportion of men with sarcopenia had osteopenia compared to non-sarcopenic men. A cross-sectional study in a geriatric inpatient population found that prevalence of osteoporosis was significantly higher in men with sarcopenia than in men without sarcopenia [36]. Older Chinese men with sarcopenia, diagnosed according to Asian Working Group for Sarcopenia guidelines, had higher prevalence of osteopenia than non-sarcopenic men [37]. Furthermore, it is not only the link between bone and muscle that is of interest, but also the relationship between osteoporosis and sarcopenia, diseases which, respectively, affect bone and muscle. As mentioned in the introduction, bone and muscle are not only connected on the physical level, but a deeper molecular or genetic connection is thought to exist [38]. Therefore, it is possible that with aging, bone and muscle are both affected together rather than alone [39]. Even the term “osteosarcopenia” is used to describe people with osteoporosis and sarcopenia [18]. 

Our study has some limitations. First, the number of study participants is probably not large enough to show significant relationships between certain variables. Second, muscle fibers from the microbiopsy cannot be ideally orientated in the same cutting direction (e.g., perpendicular), which gives additional variation in morphometry (fiber area, perimeter). This explains why there was no correlation between muscle morphology and bone density.

As this was a pilot study and analysis was made based on a small sample size, future research could focus on assessing the relationship between muscle morphology and physical performance as associations between muscle fiber and the balance test was found. Finally, the inclusion of women in the study could impact on future results.

## 5. Conclusions

The results of our study show that in sarcopenic men, lower lean muscle mass was associated with lower muscle strength, physical performance and with lower femoral neck BMD. In men with osteosarcopenia, low scores on the balance test were associated with low muscle fiber area.

## Figures and Tables

**Table 1:** Basic descriptive characteristics of the study population (mean ± SD).

Characteristics	All Subjects (*n* = 151)	No Sarcopenia (*n* = 106)	Sarcopenia (*n* = 45)	*p*-Value *
Age, years	72.9 ± 8.02	70.33 ± 6.47	79.09 ± 8.05	<0.001
Height, cm	172.73 ± 6.73	173.56 ± 6.56	170.74 ± 6.79	0.006
Weight, kg	81.5 ± 13.85	84.56 ± 13.44	74.15 ± 12.05	<0.001
BMI, kg/m^2^	27.24 ± 4.07	28.01 ± 4	25.4 ± 3.66	0.001
Total fat mass, kg	24.75 ± 8.83	25.86 ± 8.87	21.99 ± 7.91	0.01
Android fat mass, kg (median (IQR))	2.57 (1.8–3.22)	2.71 (2.03–3.44)	2.3 (1.5–3.04)	0.023
Gynoid fat mass, kg (median (IQR))	3.35 (2.77–4.06)	3.5 (2.96–4.17)	3.06 (2.27–3.67)	0.006
Lean mass, kg	54.01 ± 6.61	55.88 ± 6.06	49.37 ± 5.7	<0.001
Appendicular lean mass, kg	24.32 ± 3.68	25.5 ± 3.34	21.51 ± 2.88	<0.001
Arm lean mass, kg	6.78 ± 1.12	7.12 ± 1	5.85 ± 0.9	<0.001
Leg lean mass, kg	17.61 ± 2.69	18.42 ± 2.47	15.66 ± 2.13	<0.001
SMMI, aSM/m^2^	8.13 ± 0.94	8.44 ± 0.82	7.37 ± 0.78	<0.001
Handgrip strength, kg	31.54 ± 10.37	35.76 ± 8.66	21.39 ± 6.32	<0.001
Balance test, s (median (IQR))	10 (10–10)	10 (10–10)	10 (7.65–10)	<0.001
5 chair stands, s	15.18 ± 5.45	14.18 ± 4.1	17.17 ± 7.61	0.017
Gait speed, m/s (median (IQR))	0.88 (0.72–1)	1 (0.85–1.08)	0.66 (0.57–0.77)	<0.001
SPPB, score (median (IQR))	9 (8–9)	9 (8–9)	8 (7.5–8.5)	<0.001
Whole body BMD, g/cm^2^	1.19 ± 0.14	1.22 ± 0.13	1.11 ± 0.14	0.001
Lumbar spine BMD, g/cm^2^	1.22 ± 0.23	1.2 ± 0.23	1.17 ± 0.33	0.157
Femoral neck BMD, g/cm^2^	0.92 ± 0.15	0.97 ± 0.14	0.83 ± 0.12	<0.001
Whole body T-score (median (IQR))	−0.1 (−1–0.85)	0.1 (−0.7–1.1)	−1.1 (−1.7–0.1)	<0.001
Lumbar spine BMD T-score (median (IQR))	−0.4 (−1.3–1.3)	−0.4 (−1.2–1.3)	−0.6 (−1.55–0.6)	0.17
Femoral neck BMD T-score (median (IQR))	−1.2 (−1.9–−0.4)	−0.9 (−1.6–−0.1)	−1.9 (−2.57–−1.3)	<0.001
T-score ≤ −1.0, number (%)	97 (64.2)	58 (54.7)	39 (86.7)	<0.001
Whole body BMC, kg	3 ± 0.47	3.11 ± 0.44	2.73 ± 0.45	<0.001
Number of comorbidities (median (IQR))	1 (1–2)	1 (1–2)	1 (1–2)	0.503
Number of medications (median (IQR))	1 (1–2)	1 (1–2)	1.5 (1–3)	0.277

BMI—body mass index, SMMI—skeletal muscle mass index, aSM—appendicular skeletal muscle mass, IQR—interquartile range, BMD—bone mineral density, BMC—bone mineral content, SPPB—short physical performance battery, * *p*-value comparing non-sarcopenic and sarcopenic groups.

**Table 2:** Correlation coefficient (r) between bone density, body composition and physical performance parameters among sarcopenic men.

Lean Mass Parameters	Bone Density Measures			
	Whole Body BMD, g/cm^2^	Femoral Neck BMD, g/cm^2^	Whole Body T-Score	Femoral Neck T-Score
Lean mass, kg	0.37 *	0.418 *	0.37 *	0.446 *
Appendicular lean mass, kg	0.44 *	0.5 *	0.442 *	0.52 *
Arm lean mass, kg	0.451 *	0.576 *	0.457 *	0.564 *
Leg lean mass, kg	0.382 *	0.435 *	0.382 *	0.411 *
SMMI, aSM/m^2^	0.382 *	0.347 *	0.372 *	0.362 *
Physical performance				
SPPB, score	0.57	0.223 *	0.082	0.219 *
Gait speed, m/s	0.14	0.301	0.05	0.46 *
Balance test, s	0.024	0.442 *	0.368 *	0.45 *

SMMI—skeletal muscle mass index, aSM—appendicular skeletal muscle mass, BMD—bone mineral density, SPPB—short physical performance battery, r—Spearman’s coefficient, * *p* < 0.05.

## Data Availability

The data presented in this study are available on request from the corresponding author. The data are not publicly available due to ethical reasons.

## References

[1] Rosenberg I.H. (1989). Summary comments. Am. J. Clin. Nutr..

[2] Morley J., Anker S., von Haehling S. (2014). Prevalence, incidence, and clinical impact of sarcopenia: Facts, numbers, and epidemiology-update 2014. J. Cachexia Sarcopenia Muscle.

[3] Mayhew A.J., Amog K., Phillips S., Parise G., McNicholas P.D., de Souza R.J., Thabane L., Raina P. (2018). The prevalence of sarcopenia in community-dwelling older adults, an exploration of differences between studies and within definitions: A systematic review and meta-analyses. Age Ageing.

[4] Veronese N., Demurtas J., Soysal P., Smith L., Torbahn G., Schoene D., Schwingshackl L., Sieber C., Bauer J., Cesari M. (2019). Sarcopenia and health-related outcomes: An umbrella review of observational studies. Eur. Geriatr. Med..

[5] Beaudart C., Zaaria M., Pasleau F., Reginster J.-Y., Bruyère O. (2017). Health Outcomes of Sarcopenia: A Systematic Review and Meta-Analysis. PLoS ONE.

[6] Lexell J., Henriksson-Larsén K., Winblad B., Sjöström M. (1983). Distribution of different fiber types in human skeletal muscles: Effects of aging studied in whole muscle cross sections. Muscle Nerve.

[7] Deschenes M.R. (2004). Effects of Aging on Muscle Fibre Type and Size. Sports Med..

[8] Purves-Smith F.M., Sgarioto N., Hepple R.T. (2014). Fiber typing in aging muscle. Exerc. Sport Sci. Rev..

[9] Toomey C.M., Cremona A., Hughes K., Norton C., Jakeman P. (2015). A Review of Body Composition Measurement in the Assessment of Health. Top. Clin. Nutr..

[10] Compston J.E., McClung M.R., Leslie W.D. (2019). Osteoporosis. Lancet.

[11] Ho-Pham L.T., Nguyen U.D.T., Nguyen T.V. (2014). Association between Lean Mass, Fat Mass, and Bone Mineral Density: A Meta-analysis. J. Clin. Endocrinol. Metab..

[12] Westbury L.D., Syddall H.E., Fuggle N.R., Dennison E.M., Cauley J.A., Shiroma E.J., Fielding R.A., Newman A.B., Cooper C. (2020). Long-term rates of change in musculoskeletal aging and body composition: Findings from the Health, Aging and Body Composition Study. Calcif. Tissue Int..

[13] Kim K.M., Lim S., Oh T.J., Moon J.H., Choi S.H., Lim J.Y., Kim K.W., Park K.S., Jang H.C. (2017). Longitudinal Changes in Muscle Mass and Strength, and Bone Mass in Older Adults: Gender-Specific Associations Between Muscle and Bone Losses. J. Gerontol. Ser. A.

[14] Frost H.M. (1996). Perspectives: A proposed general model of the “mechanostat” (suggestions from a new skeletal-biologic paradigm). Anat. Rec..

[15] Isaacson J., Brotto M. (2014). Physiology of Mechanotransduction: How Do Muscle and Bone “Talk” to One Another?. Clin. Rev. Bone Miner. Metab..

[16] Cruz-Jentoft A.J., Baeyens J.P., Bauer J.M., Boirie Y., Cederholm T., Landi F., Martin F.C., Michel J.-P., Rolland Y., Schneider S.M. (2010). Sarcopenia: European consensus on definition and diagnosis: Report of the European Working Group on Sarcopenia in Older People. Age Ageing.

[17] Kerr C., Bottomley C., Shingler S., Giangregorio L., de Freitas H.M., Patel C., Randall S., Gold D.T. (2017). The Importance of Physical Function to People with Osteoporosis. Osteoporos. Int. J. Establ. Result Coop. Eur. Found. Osteoporos. Natl. Osteoporos. Found. USA.

[18] Hirschfeld H.P., Kinsella R., Duque G. (2017). Osteosarcopenia: Where Bone, Muscle, and Fat Collide. Osteoporos. Int..

[19] Kanis J.A., Kanis J.A. (1994). Assessment of Fracture Risk and Its Application to Screening for Postmenopausal Osteoporosis: Synopsis of a WHO Report. Osteoporos. Int..

[20] Baumgartner R.N., Koehler M.K., Gallager D., Romero L., Heymsfield S.B., Ross R.R., Garry P.J., Lindeman R.D. (1998). Epidemiology of sarcopenia among elderly in New Mexico. Am. J. Epidemiol..

[21] Lauretani F., Russo C.R., Bandinelli S., Bartali B., Cavazzini C., Di Iorio A., Corsi A.M., Rantanen T., Guralnik J.M., Ferrucci L. (2003). Age-associated changes in skeletal muscles and their effect on mobility: An operational diagnosis of sarcopenia. J. Appl. Physiol..

[22] Guralnik J.M., Simonsick E.M., Ferrucci L., Glynn R.J., Berkman L.F., Blazer D.G., Scherr P.A., Wallace R.B. (1994). A Short Physical Performance Battery Assessing Lower Extremity Function: Association with Self-Reported Disability and Prediction of Mortality and Nursing Home Admission. J. Gerontol..

[23] Phu S., Kirk B., Bani Hassan E., Vogrin S., Zanker J., Bernardo S., Duque G. (2020). The diagnostic value of the Short Physical Performance Battery for sarcopenia. BMC Geriatr..

[24] Deyo R.A., Diehr P., Patrick D.L. (1991). Reproducibility and responsiveness of health status measures statistics and strategies for evaluation. Control. Clin. Trials.

[25] Verschueren S., Gielen E., O’Neill T.W., Pye S.R., Adams J.E., Ward K.A., Wu F.C., Szulc P., Laurent M., Claessens F. (2013). Sarcopenia and its relationship with bone mineral density in middle-aged and elderly European men. Osteoporos. Int..

[26] Patel H.P., Dawson A., Westbury L.D., Hasnaoui G., Syddall H.E., Shaw S., Sayer A.A., Cooper C., Dennison E.M. (2018). Muscle Mass, Muscle Morphology and Bone Health Among Community-Dwelling Older Men: Findings from the Hertfordshire Sarcopenia Study (HSS). Calcif. Tissue Int..

[27] Hars M., Biver E., Chevalley T., Herrmann F., Rizzoli R., Ferrari S., Trombetti A. (2016). Low Lean Mass Predicts Incident Fractures Independently From FRAX: A Prospective Cohort Study of Recent Retirees. J. Bone Miner. Res..

[28] Intriago M., Maldonado G., Guerrero R., Messina O.D., Rios C. (2020). Bone Mass Loss and Sarcopenia in Ecuadorian Patients. J. Aging Res..

[29] Ma Y., Fu L., Jia L., Han P., Kang L., Yu H., Chen X., Yu X., Hou L., Wang L. (2018). Muscle strength rather than muscle mass is associated with osteoporosis in older Chinese adults. J. Formos. Med Assoc..

[30] Liu P.-Y., Ilich J.Z., Brummel-Smith K., Ghosh S. (2014). New insight into fat, muscle and bone relationship in women: Determining the threshold at which body fat assumes negative relationship with bone mineral density. Int. J. Prev. Med..

[31] Lee I., Cho J., Jin Y., Ha C., Kim T., Hyunsik K. (2016). Body Fat and Physical Activity Modulate the Association between Sarcopenia and Osteoporosis in Elderly Korean Women. J. Sports Sci. Med..

[32] Qi H., Sheng Y., Chen S., Wang S., Zhang A., Cai J., Lai B., Ding G. (2019). Bone mineral density and trabecular bone score in Chinese subjects with sarcopenia. Aging Clin. Exp. Res..

[33] Lindblad A., Dahlin-Ivanoff S., Bosaeus I., Rothenberg E. (2015). Body Composition and Hand Grip Strength in Healthy Community-dwelling Older Adults in Sweden. J. Aging Res. Clin. Pract..

[34] Charlton K., Batterham M., Langford K., Lateo J., Brock E., Walton K., Lyons-Wall P., Eisenhauer K., Green N., McLean C. (2015). Lean Body Mass Associated with Upper Body Strength in Healthy Older Adults While Higher Body Fat Limits Lower Extremity Performance and Endurance. Nutrients.

[35] Terracciano C., Celi M., Lecce D., Baldi J., Rastelli E., Lena E., Massa R., Tarantino U. (2013). Differential features of muscle fiber atrophy in osteoporosis and osteoarthritis. Osteoporos. Int..

[36] Reiss J., Iglseder B., Alzner R., Mayr-Pirker B., Pirich C., Kässmann H., Kreutzer M., Dovjak P., Reiter R. (2019). Sarcopenia and osteoporosis are interrelated in geriatric inpatients. Z. Gerontol. Geriatr..

[37] Yu R., Leung J., Woo J. (2014). Incremental Predictive Value of Sarcopenia for Incident Fracture in an Elderly Chinese Cohort: Results From the Osteoporotic Fractures in Men (MrOs) Study. J. Am. Med Dir. Assoc..

[38] Avin K.G., Bloomfield S.A., Gross T.S., Warden S.J. (2015). Biomechanical Aspects of the Muscle-Bone Interaction. Curr. Osteoporos. Rep..

[39] Reginster J.-Y., Beaudart C., Buckinx F., Bruyère O. (2016). Osteoporosis and Sarcopenia: Two Diseases or One?. Curr. Opin. Clin. Nutr. Metab. Care.

